# YAP prevents premature senescence of astrocytes and cognitive decline of Alzheimer's disease through regulating CDK6 signaling

**DOI:** 10.1111/acel.13465

**Published:** 2021-08-20

**Authors:** Xingxing Xu, Xiya Shen, Jiaojiao Wang, Wenjin Feng, Mianxian Wang, Xuemeng Miao, Qian Wu, Lihao Wu, Xiaoning Wang, Yimin Ma, Shuang Wu, Xiaomei Bao, Wei Wang, Ying Wang, Zhihui Huang

**Affiliations:** ^1^ School of Basic Medical Sciences Wenzhou Medical University Wenzhou China; ^2^ Zhejiang Sinogen Medical Equipment Co., Ltd Wenzhou China; ^3^ School of Mental Health Wenzhou Medical University Wenzhou China; ^4^ School of the First Clinical Medical Sciences School of Information and Engineering Wenzhou Medical University Wenzhou China; ^5^ Department of Obstetrics and Gynecology Wenzhou People's Hospital Wenzhou China; ^6^ Phase I Clinical Research Center Zhejiang Provincial People's Hospital of Hangzhou Medical College Hangzhou China; ^7^ College of Pharmacy Hangzhou Normal University Hangzhou China

**Keywords:** alzheimer's disease, astrocytes, CDK6, senescence, YAP

## Abstract

Senescent astrocytes accumulate with aging and contribute to brain dysfunction and diseases such as Alzheimer's disease (AD), however, the mechanisms underlying the senescence of astrocytes during aging remain unclear. In the present study, we found that Yes‐associated Protein (YAP) was downregulated and inactivated in hippocampal astrocytes of aging mice and AD model mice, as well as in D‐galactose and paraquat‐induced senescent astrocytes, in a Hippo pathway‐dependent manner. Conditional knockout of YAP in astrocytes significantly promoted premature senescence of astrocytes, including reduction of cell proliferation, hypertrophic morphology, increase in senescence‐associated β‐galactosidase activity, and upregulation of several senescence‐associated genes such as p16, p53 and NF‐κB, and downregulation of Lamin B1. Further exploration of the underlying mechanism revealed that the expression of cyclin‐dependent kinase 6 (CDK6) was decreased in YAP knockout astrocytes in vivo and in vitro, and ectopic overexpression of CDK6 partially rescued YAP knockout‐induced senescence of astrocytes. Finally, activation of YAP signaling by XMU‐MP‐1 (an inhibitor of Hippo kinase MST1/2) partially rescued the senescence of astrocytes and improved the cognitive function of AD model mice and aging mice. Taken together, our studies identified unrecognized functions of YAP‐CDK6 pathway in preventing astrocytic senescence in vitro and in vivo, which may provide further insights and new targets for delaying brain aging and aging‐related neurodegenerative diseases such as AD.

## Graphical abstract

YAP signaling is downregulated and inactivated in hippocampal astrocytes of aging mice and AD model mice, as well as in D‐gal‐ and PQ‐induced senescent astrocytes, in a Hippo pathway‐dependent manner. Mechanistically, YAP prevents the premature senescence of astrocytes through CDK6 signaling. Activation of YAP by XMU‐MP‐1 delays the senescence of astrocytes, and thereby improving the cognitive function of aged mice and AD model mice.

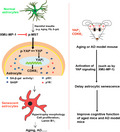

**Abbreviations**
AD: Alzheimer's disease
CDK6: cyclin‐dependent kinase 6
D‐gal: D‐galactose
PD: Parkinson's disease
PQ: paraquat
SA‐β‐Gal: senescence‐associated β‐galactosidase
WT: wild‐type
YAP: Yes‐associated Protein

## 1 INTRODUCTION

As one of the most important body organs, the brain is particularly sensitive to aging (Yankner et al., 2008). Brain aging is accompanied by a decline in various cognitive functions (Yankner et al., 2008). In addition, the risk for aging‐related neurodegenerative diseases, such as Alzheimer's disease (AD) (Ziegler‐Graham et al., 2008) and Parkinson's disease (PD) (Myall et al., 2017; Reeve et al., 2014), exponentially increase with age. Therefore, how to delay brain aging and prevent aging‐related diseases is critical for human health.

Brain aging begins with the senescence of cells in the brain. Recent studies have shown that astrocytic specific genes alter their expression patterns in human hippocampus during aging (Soreq et al., 2017). Interestingly, astrocytic senescence has been proposed as a component of AD (Bhat et al., 2012; Garwood et al., 2017). Clearance of these senescent glial cells prevents tau‐dependent pathology and cognitive hypofunction (Bussian et al., 2018). Senescent astrocytes usually exhibit large, flat, and vacuolated cell morphology, elevated senescence‐associated β‐galactosidase (SA‐β‐Gal) activity, attenuated cell proliferation and accumulation of p16 (Chinta et al., 2018; Y. Shen et al., 2014). Although some molecules such as glutamine synthetase (Y. Shen et al., 2014), p38MAPK (Mombach et al., 2015), angiotensin II (Liu et al., 2011), and Δ133p53 (Turnquist et al., 2019) are involved in the regulation of astrocytic senescence, it remains largely unclear that the molecular mechanism underlying astrocytic senescence.

The Hippo signaling pathway is a key regulator of stem cell self‐renewal, tissue regeneration and organ size, and is a conserved classical pathway formed by the kinase cascade (F. X. Yu et al., 2013; F. X. Yu et al., 2015; F. X. Yu et al., 2012). As a key effector of the Hippo signaling pathway, YAP has been shown to play an important roles in cell proliferation, differentiation and tissue regeneration (Bao et al., 2020; Rosado‐Olivieri et al., 2019; X. Shen et al., 2020; C. Xie et al., 2020). Recent evidence has shown that YAP signaling is closely related to individual aging and cellular senescence (Fu et al., 2019; Iwasa et al., 2013; Jia et al., 2018; Jin et al., 2017; Santinon et al., 2018; Q. Xie et al., 2013; X. Xu et al., 2020). YAP‐1 deficiency promotes health aging of *Caenorhabditis elegans* (Iwasa et al., 2013), and YAP inhibits the senescence of human fibroblast cells (Santinon et al., 2018; Q. Xie et al., 2013), human mesenchymal stem cells (Fu et al., 2019), human periodontal ligament stem cells (Jia et al., 2018), glioma cells (X. Xu et al., 2020), and hepatic stellate cells (Jin et al., 2017). However, it remains unclear whether YAP signaling regulates the senescence of astrocytes in aged brain and AD.

In the present study, we found that YAP was downregulated and inactivated in senescent astrocytes in vitro and in vivo, and YAP prevented the senescence of astrocytes through the CDK6 signaling. Our study provides a new molecular mechanism for regulating astrocytic senescence, which may provide new insights and targets for delaying brain aging and aging‐related neurodegenerative diseases.

## 2 RESULTS

### 2.1 YAP is downregulated and inactivated in hippocampal astrocytes of aged mice and AD model mice

**FIGURE 1:**
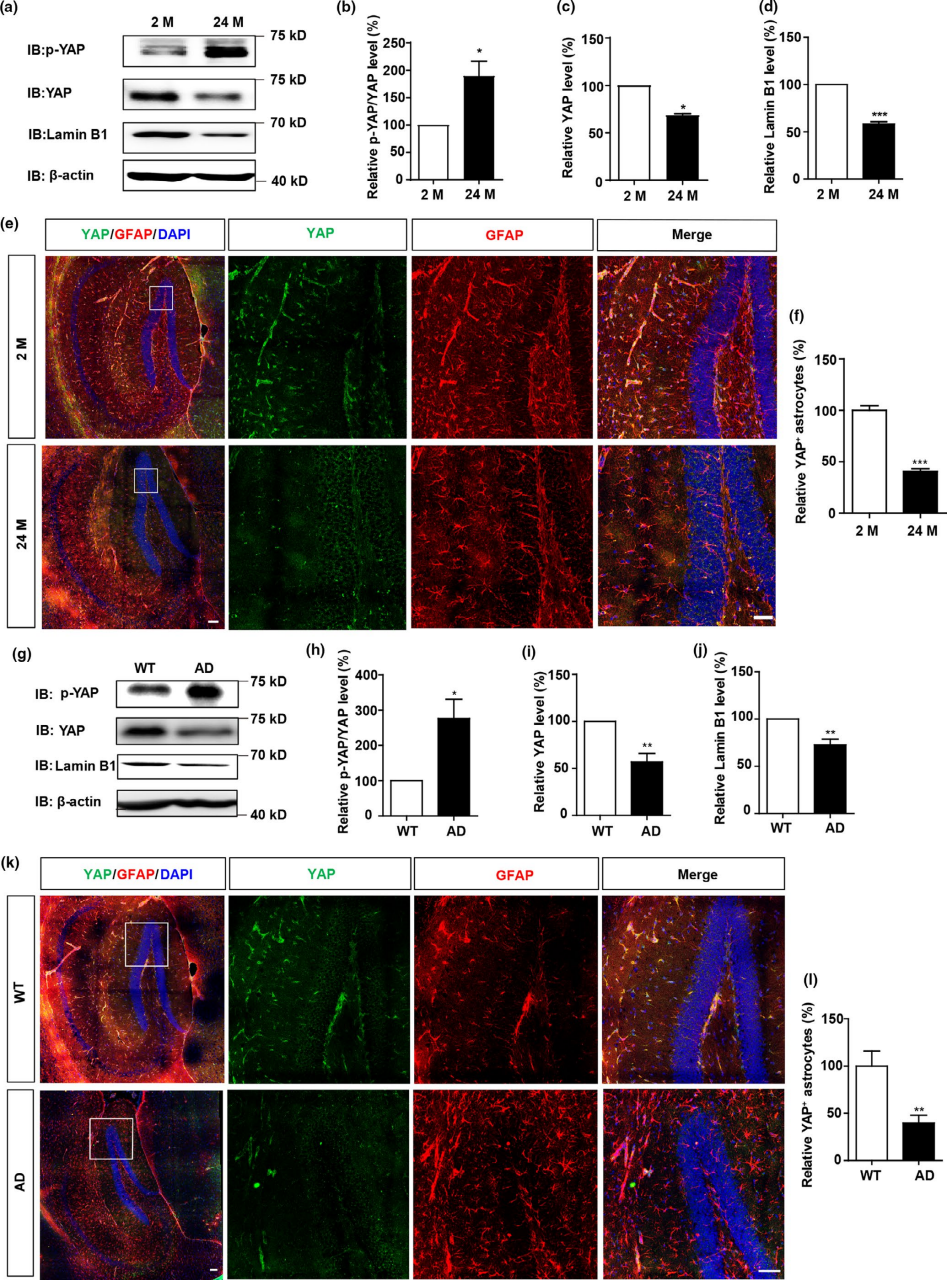
YAP is downregulated and inactivated in hippocampal astrocytes of aged mice and AD model mice. (a) Western blot analysis of p‐YAP, YAP, and Lamin B1 expression in the hippocampus of 2 M and 24 M mice. (b–d) Quantification of relative expression of p‐YAP/YAP (b), YAP (c) and Lamin B1 (d) as shown in (a) (n = 3 per group, normalized to 2 M mice). (e) Double immunostaining analysis of YAP (green) and GFAP (red) in the hippocampus of 2 M and 24 M mice. (f) Quantitative analysis of YAP^+^ cells over total astrocytes as shown in (e) (n = 3, normalized to 2 M mice). (g) Western blot analysis of p‐YAP, YAP, and Lamin B1 expression in the hippocampus of WT and AD model mice (6 M). (h–j) Quantification of relative expression of p‐YAP/YAP (h), YAP (i) and Lamin B1 (j) as shown in (g) (n = 3, normalized to WT mice). (k) Double immunostaining analysis of YAP (green) and GFAP (red) in the hippocampus of WT and AD model mice (6 M). (l) Quantitative analysis of YAP^+^ cells over total astrocytes as shown in (k) (n = 3, normalized to WT mice). Scale bar, 100 μm. Data were mean ± s.e.m. *p < 0.05, **p < 0.01, ***p < 0.001

To explore the potential functions of YAP in the aging brain, hippocampal tissues of 2 M and 24 M old mice were collected. SA‐β‐gal staining, which is a classical method for testing cell or tissue senescence (Dimri et al., 1995), showed the increased β‐gal intensity in hippocampus of 24 M old mice, compared with that in 2 M old mice (Figure S1a). In these hippocampus of 24 M old mice, the protein levels of YAP were significantly decreased, as well as the decrease in Lamin B1 (a senescent marker) levels, which is a common feature of senescent cells (Freund et al., 2012; Shimi et al., 2011), however, both p‐YAP and p‐YAP/YAP ratio were significantly increased (Figure 1a–d). Moreover, the phosphorylation levels of the Hippo kinases such as LATS1, MST1, MOB1 and YAP were increased significantly in old mice, compared with that in young mice (Figure S1b–h). These results suggest that YAP is downregulated and inactivated in aged brains in a Hippo pathway‐dependent manner. Our previous studies have shown that YAP is mainly expressed in astrocytes and neural stem cells of the brain (Z. Huang et al., 2016). Indeed, as shown in Figure 1e–f, the expression of YAP was dramatically decreased in the GFAP positive astrocytes of 24 M old hippocampus.

To further confirm whether YAP is also downregulated and inactivated in aging‐related diseases such as AD, YAP expression was detected in the hippocampal tissues of APP/PS1 mice (Figure S1i), a transgenic mouse model of AD (Lok et al., 2013). As shown in Figure 1g–j, again, both YAP and Lamin B1 expression were decreased, however, p‐YAP levels and the p‐YAP/YAP ratios were increased in the hippocampus tissues of AD model mice. The phosphorylation levels of the Hippo kinases such as LATS1, MST1, MOB1 and YAP were elevated significantly in the AD model mice, compared with that in wild‐type (WT) mice (Figure S1j–p), implying that the downregulation and inactivation of YAP in AD model mice was dependent on the Hippo pathway. Moreover, immunohistochemical staining also showed that YAP expression was suppressed in the hippocampal astrocytes of AD model mice (Figure 1k,l). Taken together, these results indicate that YAP is downregulated and inactivated in the hippocampal astrocytes of aged mice and AD model mice, which may contribute to brain aging.

### 2.2 YAP is downregulated and inactivated in the naturally senescent astrocytes and D‐galactose (D‐gal) or paraquat (PQ)‐induced senescent astrocytes in vitro

**FIGURE 2:**
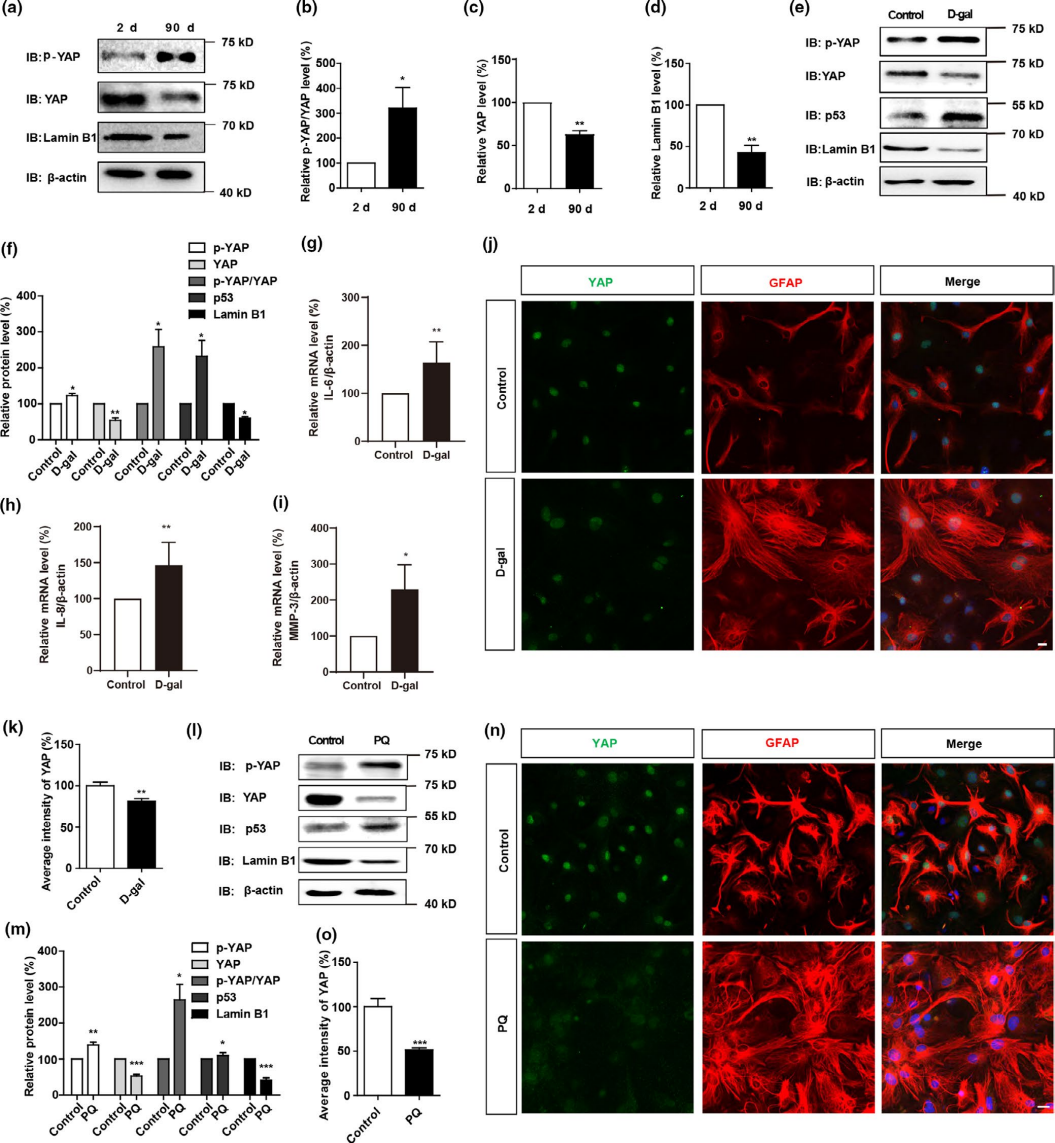
YAP is downregulated and inactivated both in naturally senescent astrocytes and D‐gal or PQ‐induced senescent astrocytes. (a) Western blot analysis of p‐YAP, YAP and Lamin B1 expression in primary cultured astrocytes at DIV 2 and 90. (b–d) Quantification of relative expression of p‐YAP/YAP (b), YAP (c) and Lamin B1 (d) as shown in (a) (n = 3, normalized to astrocytes at DIV 2). (e) Western blot analysis of p‐YAP, YAP, p53, and Lamin B1 expression in young astrocytes and senescent astrocytes. (f) Quantification of relative p‐YAP, YAP, p‐YAP/YAP, p53 and Lamin B1 expression as shown in (e) (n = 3 per group, normalized to control astrocytes). (g–i) qPCR analysis of relative mRNA levels of IL‐6 (g, n = 4), IL‐8 (h, n = 3) and MMP‐3 (i, n = 3) (normalized to control astrocytes) in control and D‐gal‐induced senescent astrocytes. (j) Double immunostaining analysis of YAP (green) and GFAP (red) in control and D‐gal‐induced senescent astrocytes. (k) Quantitative analysis of the relative average intensity of YAP as shown in (j) (n = 15, normalized to control astrocytes). (l) Western blot analysis of p‐YAP, YAP, p53 and Lamin B1 expression in control and PQ‐induced senescent astrocytes. (m) Quantification of relative expression of p‐YAP, YAP, p‐YAP/YAP, p53 and Lamin B1 as shown in (l) (n = 3 per group, normalized to control astrocytes). (n) Double immunostaining analysis of YAP (green) and GFAP (red) in the control and PQ‐induced senescent astrocytes. (o) Quantitative analysis of the relative average intensity of YAP as shown in (n) (n = 15, normalized to control astrocytes). Scale bar, 20 μm. Data were mean ± s.e.m. *p < 0.05, **p < 0.01, ***p < 0.001

To further examine the expression pattern of YAP in senescent astrocytes, the primary astrocytes were cultured to DIV 90, which is considered as naturally senescent astrocytes (Pertusa et al., 2007), interestingly, in these naturally senescent astrocytes, both Lamin B1 and YAP were significantly decreased, however, p‐YAP and p‐YAP/YAP levels were increased (Figure 2a–d). To further investigate the functions of YAP in pathological senescent astrocytes, D‐gal‐induced senescent astrocyte models (Y. Shen et al., 2014) were used. In our system, primary cultured astrocytes were treated with D‐gal at 111 mM for 5 days, and then SA‐β‐gal staining confirmed the senescence of astrocytes (Figure S2a–c). Moreover, mRNA levels of p16 (a senescent marker) were significantly increased in these senescent astrocytes (Figure S2d). In addition, these astrocytes showed hypertrophic morphology, reduction of cell proliferation and decrease in Lamin B1 expression (Figure S2e–h), which all indicate that D‐gal induces the senescence of astrocytes. Moreover, consistent with our results in vivo, YAP expression was significantly downregulated, however, p‐YAP level and p‐YAP/YAP ratio were increased in these D‐gal‐induced senescent astrocytes, as well as increase in p53 expression (a senescent marker) and decrease in Lamin B1 expression (Figure 2e–f). qPCR analysis showed that the secretion of SASP factors, such as IL‐6, IL‐8 and MMP‐3, were increased in senescent astrocytes (Figure 2g–i). Furthermore, immunostaining also showed that YAP level was decreased in these senescent astrocytes (Figure 2j–k). These results suggest that YAP is downregulated and inactivated in D‐gal‐induced senescent astrocytes.

To further confirm our results, another senescent astrocyte model was established as reported previously (Chinta et al., 2018). Young astrocytes were treated with PQ for 24 h, then, PQ was removed from the culture medium, and the astrocytes were recovered for another 8 days (Figure S2i). As shown in Figure S2j–l, SA‐β‐gal staining revealed that the percentage of β‐galactosidase positive astrocytes was elevated significantly after PQ treatment, while the expression of Lamin B1 was significantly decreased, which indicates that PQ could induce the senescence of astrocytes. Similarly, in these senescent astrocytes, YAP expression was significantly reduced, whereas p‐YAP level and p‐YAP/YAP ratio were increased, as well as the increase in p53 level and decrease in Lamin B1 level (Figure 2l,m). Furthermore, immunostaining also showed decrease in YAP expression in PQ‐induced senescent astrocytes (Figure 2n,o).

To determine whether the inactivation of YAP in senescent astrocytes in vitro is dependent on activation of the Hippo pathway, the protein levels of Hippo kinases were detected. As shown in Figure S3a–g, the protein levels of LATS1, MST1, and SAV1 were decreased in senescent astrocytes, whereas the levels of p‐LATS1/LATS1, p‐MST1/MST1, and p‐MOB1 were increased in senescent astrocytes, indicating that inactivation of YAP signaling in astrocytic senescence is dependent on Hippo pathway. Moreover, mRNA levels of LATS1, MST1, and SAV1 were significantly decreased (Figure S3h–j) in senescent astrocytes, which indicated that the reduction of these proteins after senescence was probably due to suppressed transcription of these genes. Taken together, these results suggest that YAP is downregulated and inactivated both in the naturally senescent astrocytes and D‐gal‐ or PQ‐induced senescent astrocytes in a Hippo pathway‐dependent manner.

### 2.3 YAP deletion in astrocytes promotes the premature senescence of astrocytes in vitro and in vivo

To examine the roles of YAP in senescent astrocytes, YAP^GFAP^‐CKO mice (conditionally knockout YAP in astrocytes) were generated, by crossing YAP^f/f^ mice with GFAP‐cre transgenic mice. Both western blot and immunostaining showed that YAP was efficiently knockout in YAP^GFAP^‐CKO mice and cultured YAP^−/−^ astrocytes (Figure S4a–f).

**FIGURE 3:**
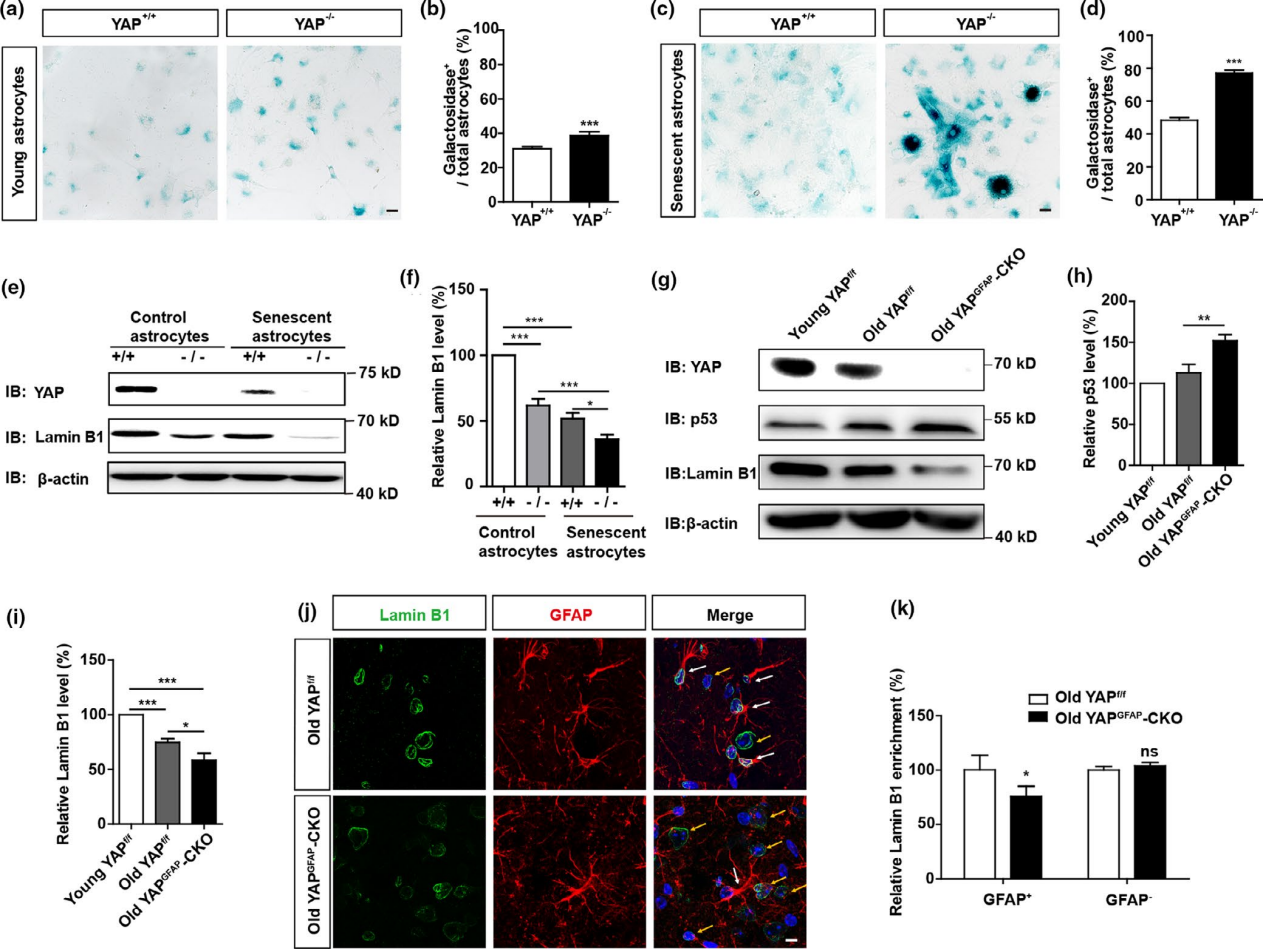
YAP deletion promotes premature senescence of astrocytes in vitro and in vivo. (a) Representative images of SA‐β‐gal staining of young YAP^+/+^ and YAP^−/−^ astrocytes. (b) Quantification of the percentage of β‐galactosidase^+^ astrocytes over total astrocytes as shown in (a) (n = 15). (c) Representative images of SA‐β‐gal staining of senescent YAP^+/+^ and YAP^−/−^ astrocytes induced by D‐gal. (d) Quantification of the percentage of β‐galactosidase^+^ astrocytes over total astrocytes as shown in (c) (n = 15). (e) Western blot detected YAP and Lamin B1 expression in YAP^+/+^ and YAP^−/−^ control astrocytes and senescent astrocytes induced by D‐gal. (f) Quantification of relative Lamin B1 expression as shown in (e) (n = 3 per group, normalized to control YAP^+/+^ astrocytes). (g) Western blot analysis of YAP, p53 and Lamin B1 protein expression in the hippocampus of young YAP^f/f^ mice (2 M), old YAP^f/f^ mice (18 M), and old YAP^GFAP^‐CKO mice (18 M). (h,i) Quantification of relative expression of p53 and Lamin B1 as shown in (g) (n = 3 per group, normalized to young YAP^f/f^ mice). (j) Double immunostaining analysis of Lamin B1 (green) and GFAP (red) in the hippocampus of old YAP^f/f^ mice (18 M), and old YAP^GFAP^‐CKO mice (18 M), respectively. (k) Quantitative analysis of the relative average intensity of Lamin B1 as shown in (j) (n = 15, normalized to old YAP^f/f^ mice). ns, not significant. Scale bar, 20 μm. Data were mean ± s.e.m. *p < 0.05, **p < 0.01, ***p < 0.001

Interestingly, SA‐β‐gal staining showed that the percentage of β‐galactosidase positive cells was increased in YAP^−/−^ astrocytes (Figure 3a,b). Moreover, YAP deletion significantly reduced the proliferation of astrocytes (Figure S5a,b) and increased the mRNA expression of p21 and MMP‐3 (Figure S5c–d). Deletion of YAP in astrocytes significantly aggravated D‐gal‐induced senescence of astrocytes (Figure 3c–d), and significantly reduced the expression of Lamin B1 in D‐gal‐induced senescent astrocytes (Figure 3e–f). Moreover, more significant increase in β‐galactosidase positive cells and more potent reduction of Lamin B1 were found in YAP^−/−^ astrocytes, which suggest that YAP^−/−^ astrocytes was more vulnerable to senescence induction, compared with YAP^+/+^ astrocytes (Figure 3a–f). These results suggest that YAP deletion promotes the premature senescence of astrocytes in vitro.

Again, in the hippocampus of old YAP^f/f^ mice, the expression of YAP and Lamin B1 was significantly decreased, and p53 expression was significantly increased, compared with that in young YAP^f/f^ mice. As expected, in YAP^GFAP^‐CKO mice, p53 expression was significantly increased than that in young YAP^f/f^ mice and old YAP^f/f^ mice, however, Lamin B1 expression was significantly decreased (Figure 3g–i). Immunohistochemistry showed that in the hippocampus of old YAP^GFAP^‐CKO mice, the expression of Lamin B1 was significantly decreased in GFAP positive astrocytes, compared to old YAP^f/f^ mice, but not in GFAP negative cells (Figure 3j–k). Moreover, in the hippocampus of old YAP^GFAP^‐CKO mice, the expression of Lamin B1 was significantly decreased in NeuN (a marker of neurons) positive cells (Figure S6a–b), but was comparable in Iba1 (a marker of the microglia) positive cells, compared to old YAP^f/f^ mice (Figure S6c–d). Taken together, these results suggest that YAP deletion promotes the premature senescence of astrocytes in vitro and in vivo.

### 2.4 YAP prevents premature senescence of astrocytes through the CDK6 pathway

**FIGURE 4:**
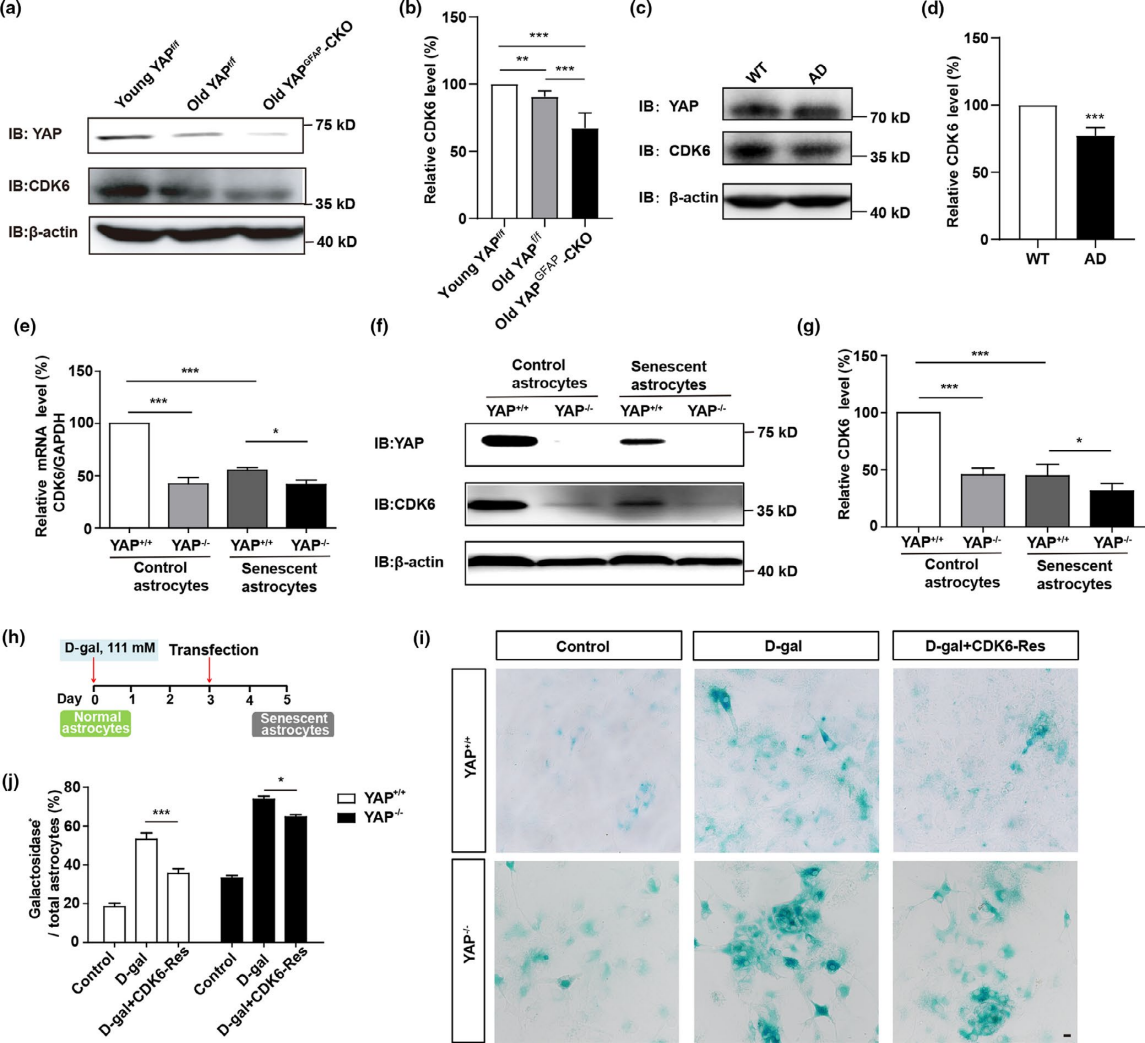
YAP prevents premature senescence of astrocytes through the CDK6 pathway. (a) Western blot analysis of YAP and CDK6 expression in the hippocampus of young YAP^f/f^ mice (2 M), old YAP^f/f^ mice (18 M), and old YAP^GFAP^‐CKO mice (18 M). (b) Quantification of relative expression of CDK6 as shown in (a) (n = 12 per group, normalized to young YAP^f/f^ mice). (c) Western blot analysis of YAP and CDK6 expression in the hippocampus of WT mice (2 M) and AD model mice (6 M). (d) Quantification of relative CDK6 expression as shown in (c) (n = 9, normalized to WT mice). (e) qPCR analysis of relative CDK6 mRNA levels in YAP^+/+^ and YAP^−/−^ control and D‐gal‐induced senescent astrocytes (n = 3 per group, normalized to control YAP^+/+^ astrocytes). (f) Western blot analysis of YAP and CDK6 expression in YAP^+/+^ and YAP^−/−^ control and D‐gal‐induced senescent astrocytes. (g) Quantification of relative CDK6 expression as shown in (f) (n = 3 per group, normalized to control YAP^+/+^ astrocytes). (h) Protocol for CDK6 transfection in D‐gal‐treated astrocytes. (i) Representative images of SA‐β‐gal staining of YAP^+/+^ and YAP^−/−^ astrocytes without transfection, or D‐gal‐induced senescent YAP^+/+^ and YAP^−/−^ astrocytes transfected with control vector or CDK6 plasmid (CDK6‐Res) for 2 days. (j) Quantification of the percentage of β‐galactosidase^+^ cells over total cells as shown in (i) (n = 15 per group). Scale bar, 20 μm. Data were mean ± s.e.m. *p < 0.05, **p < 0.01, ***p < 0.001

How does YAP prevent the senescence of astrocytes? Previous studies have reported that knockdown of YAP promotes the senescence of IMR‐90 cells through negatively regulating CDK6, which is a cyclin‐dependent kinase (Q. Xie et al., 2013), thus we tested whether YAP prevents the senescence of astrocytes through CDK6 signaling. In the hippocampus of old YAP^f/f^ mice, the expression of YAP and CDK6 were significantly decreased, compared with that in young YAP^f/f^ mice, interestingly, YAP and CDK6 expression were decreased in old YAP^GFAP^‐CKO mice, compared with that in old YAP^f/f^ mice (Figure 4a–b). Moreover, the protein levels of YAP and CKD6 were significantly decreased in AD model mice, compared with that in WT mice (Figure 4c–d). These results indicate that the YAP‐CDK6 signaling is downregulated in aged mice and AD model mice.

To further examine whether CDK6 is a target gene of YAP downstream to regulate the senescence of astrocytes, YAP^+/+^ and YAP^−/−^ astrocytes were cultured. Real‐time PCR and western blot showed that both mRNA and protein level of CDK6 were significantly decreased in YAP^−/−^ astrocytes, respectively, not only in control astrocytes, but also in D‐gal‐induced senescent astrocytes (Figure 4e–g), which suggest that YAP is required for the expression of CDK6. Furthermore, overexpression of CDK6 partially rescued D‐gal‐induced senescence of YAP^−/−^ astrocytes (Figure 4h–j). Taken together, these results suggest that YAP prevents the senescence of astrocytes through the CDK6 pathway.

### 2.5 Activation of YAP by suppressing the Hippo pathway delays the senescence of astrocytes and improves the cognitive function of aged mice and AD model mice

**FIGURE 5:**
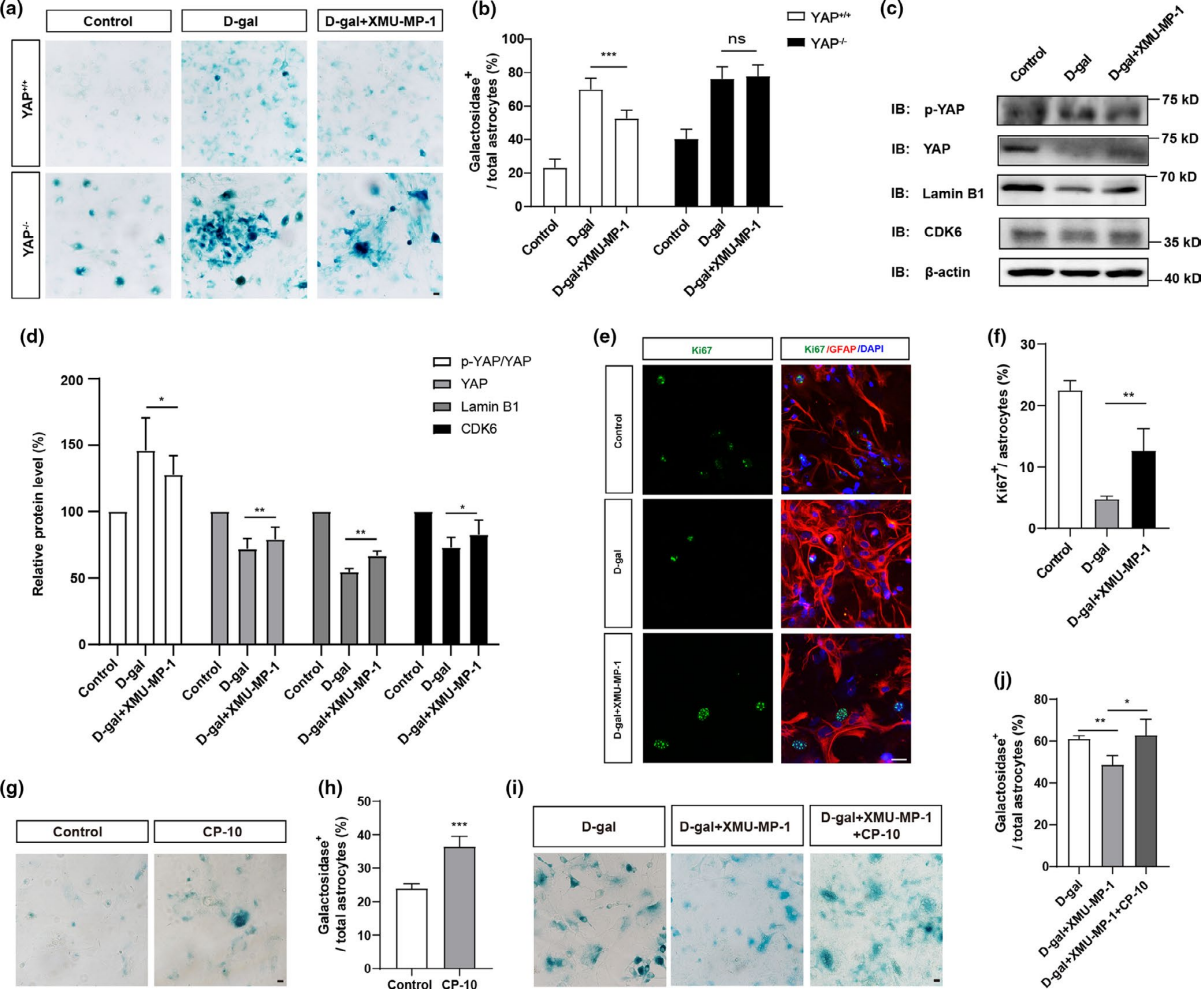
Activation of YAP by suppressing the Hippo pathway delays D‐gal‐induced senescence of astrocytes in vitro. (a) Representative images of SA‐β‐gal staining of control YAP^+/+^ and YAP^−/−^ astrocytes under various conditions. (b) Quantification of the percentage of β‐galactosidase^+^ astrocytes over total astrocytes as shown in (a) (n = 20 per group). (c) Western blot analysis of p‐YAP, YAP, Lamin B1 and CDK6 protein expression in control astrocytes, D‐gal‐induced senescent astrocytes with or without XMU‐MP‐1 treatment. (d) Quantification of relative expression of p‐YAP/YAP (n = 7), YAP (n = 7), Lamin B1 (n = 4) and CDK6 (n = 5) (normalized to control astrocytes) as shown in (c). (e) Double immunostaining analysis of Ki67 (green) and GFAP (red) in control astrocytes, D‐gal‐induced senescent astrocytes with or without XMU‐MP‐1 treatment. (f) Quantitative analysis of the percentage of Ki67^+^ astrocytes over total astrocytes as shown in (e) (n = 45 per group). (g) Representative images of SA‐β‐gal staining of cultured astrocytes with or without CP‐10 treatment. (h) Quantification of the percentage of β‐galactosidase^+^ astrocytes over total astrocytes as shown in (g) (n = 15). (i) Representative images of SA‐β‐gal staining of cultured astrocytes under various conditions. (j) Quantification of the percentage of β‐galactosidase^+^ astrocytes over total astrocytes as shown in (i) (n = 15 per group). ns, not significant. Scale bar, 20 μm. Data were mean ± s.e.m. *p < 0.05, **p < 0.01, ***p < 0.001

To investigate whether activation of YAP delays the senescence of astrocytes in vitro, XMU‐MP‐1, an inhibitor of Hippo kinase MST1/2 (Fan et al., 2016; Triastuti et al., 2019; C. Xie et al., 2020; Zhang et al., 2019), was applied. As expected, the percentage of β‐galactosidase positive cells was significantly decreased by XMU‐MP‐1 treatment in D‐gal‐induced senescence of YAP^+/+^ astrocytes, however, it failed to rescue the D‐gal‐induced senescence of YAP^−/−^ astrocytes, indicating that activation of YAP by XMU‐MP‐1 ameliorated the senescence of astrocytes (Figure 5a–b). Furthermore, western blot showed that YAP, CDK6 and Lamin B1 expression were also significantly increased, however, p‐YAP/YAP level was decreased by XMU‐MP‐1 treatment in D‐gal‐induced senescent astrocytes (Figure 5c–g). In addition, these XMU‐MP‐1‐treated astrocytes showed increased percentage of Ki67 (a marker of cell proliferation) positive astrocytes (Figure 5h–i). Interestingly, CP‐10 (a CDK6 inhibitor) promotes the senescence of astrocytes at basal level (Figure 5g,h), and the percentage of β‐galactosidase positive astrocytes in these D‐gal and XMU‐MP‐1‐treated astrocytes was elevated significantly after CP‐10 treatment (Figure 5i,j). Taken together, these results suggest that activation of YAP‐CDK6 signaling by suppressing Hippo kinases prevents the senescence of astrocytes in vitro.

**FIGURE 6:**
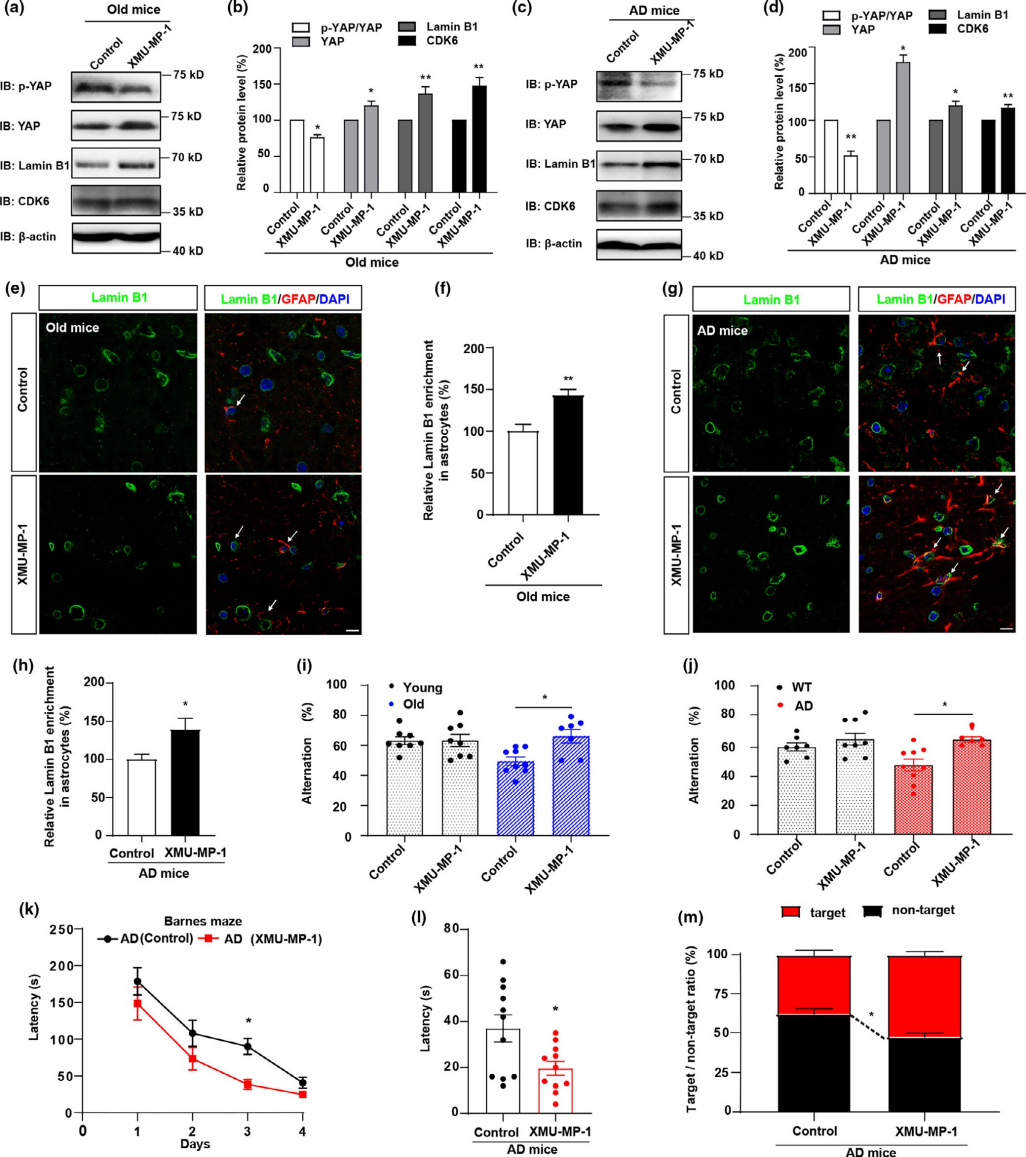
Activation of YAP by suppressing the Hippo pathway improves the cognitive function of aged mice and AD model mice. (a) Western blot analysis of p‐YAP, YAP, CDK6 and Lamin B1 protein expression in the hippocampus of old mice (18 M) with or without XMU‐MP‐1 treatment. (b) Quantification of the relative expression of p‐YAP/YAP, YAP, Lamin B1 and CDK6 as shown in (a) (n = 3 per group, normalized to control treatment). (c) Western blot analysis of p‐YAP, YAP, CDK6 and Lamin B1 protein expression in the hippocampus of AD model mice (6 M) with or without XMU‐MP‐1 treatment. (d) Quantification of the relative expression of p‐YAP/YAP, YAP, Lamin B1 and CDK6 as shown in (c) (n = 3 per group, normalized to control treatment). (e) Double immunostaining analysis of Lamin B1 (green) and GFAP (red) in the hippocampus of old mice (18 M) with or without XMU‐MP‐1 treatment. (f) Quantification of Lamin B1^+^ and GFAP^+^ cells over GFAP^+^ cells as shown in (e) (n = 10, normalized to control treatment). (g) Double immunostaining analysis of Lamin B1 (green) and GFAP (red) in the hippocampus of AD model mice (6 M) with or without XMU‐MP‐1 treatment. (h) Quantification of Lamin B1^+^ and GFAP^+^ cells over GFAP^+^ cells as shown in (g) (n = 10, normalized to control treatment). (i, j) The spontaneous alternation test conducted in WT mice (2 M) and young mice (2 M) (i), old mice (18 M) and AD model mice (6 M) (j) with or without XMU‐MP‐1 treatment by using the Y‐maze (n = 6 mice per group). (k) The time spent to reach the target exit by AD model mice with or without XMU‐MP‐1 treatment in the Barnes maze test from day 1 to day 4 (n = 6 mice). (l) The time spent to reach the target exit by AD model mice with or without XMU‐MP‐1 treatment in the Barnes maze test at the last day (n = 6 mice). (m) The target/non‐target ratio of AD model mice with or without XMU‐MP‐1 treatment (n = 6). Scale bar, 20 μm. Data were mean ± s.e.m. *p < 0.05, **p < 0.01

Subsequently, we tested whether activation of YAP restores the senescence of astrocytes in vivo, and thereby improving the cognitive function of aged mice and AD model mice. As expected, in the hippocampus of old mice and AD model mice, western blot showed that YAP, CDK6 and Lamin B1 expression were significantly upregulated, and p‐YAP/YAP level was downregulated by application of XMU‐MP‐1 (Figure 6a–d). Double immunostaining showed that the expression of Lamin B1 was significantly increased in astrocytes in old mice and AD model mice by XMU‐MP‐1 treatment (Figure 6e–h). These results suggest that activation of YAP by suppressing the Hippo pathway delays the senescence of astrocytes in vivo.

We next examined whether delay of the senescence of astrocytes improves the cognitive function of aged mice and AD model mice. Indeed, Y‐maze test showed that the percentage number of alternations was significantly increased by XMU‐MP‐1 treatment in AD model mice and old mice, but not in the WT mice or young mice (Figure 6i–j), which suggest that activation of YAP in astrocytes improves the function of learning and memory in AD model mice and old mice. Barnes maze test was further performed in AD model mice showed that the time spent to reach the target exit was reduced by XMU‐MP‐1 treatment, and the target/non‐target ratio was increased (Figure 6k–m), indicating that the cognitive function was improved in AD model mice by XMU‐MP‐1 treatment. Taken together, these results suggest that activation of YAP‐CDK6 signaling by XMU‐MP‐1 delays the senescence of astrocytes in vitro and in vivo, and thereby improving the cognitive function of aged mice and AD model mice.

## 3 DISCUSSION

In the present study, we found that YAP signaling was downregulated and inactivated through activation of Hippo kinases in senescent astrocytes in vitro and in vivo. Mechanistically, YAP prevented premature senescence of astrocytes through the CDK6 signaling. Activation of YAP signaling by XMU‐MP‐1 treatment delayed the senescence of astrocytes in vitro and in vivo, and thereby improving the cognitive function of aged mice and AD model mice (see Graphical abstracts). Our study provides a new molecular mechanism for regulating astrocytes senescence, which may provide new targets and directions for delaying brain aging and neurodegenerative diseases.

Previous studies have shown that YAP is downregulated in IMR90 cells during senescence and knockdown of YAP promotes premature senescence of IMR90 cells (Q. Xie et al., 2013), and accelerates the senescence of human mesenchymal stem cells (Fu et al., 2019) and hepatocytes (Jin et al., 2017). Consistent with these previous results, we found that YAP was downregulated and inactivated in senescent astrocytes in vivo and in vitro in a Hippo pathway‐dependent manner. We also note that one previous study has shown that the nuclear YAP was remarkably decreased in neurons under AD pathology due to the sequestration into cytoplasmic amyloid beta aggregates, which indicates YAP‐dependent neuronal necrosis in AD (Tanaka et al., 2020). However, in our studies, we found that YAP was mainly expressed in astrocytes, which was consistent with previous studies (L. Huang et al., 2020; Z. Huang et al., 2016; Z. Huang et al., 2016; C. Xie et al., 2020; H. Yu et al., 2020). The different observations may result from different samples (human patients versus mouse) or brain regions (occipital lobe of cortex versus hippocampus) or different YAP antibody (sc‐15407, Santa Cruz Biotechnology versus ab205270, Abcam or WH0010413M1, Sigma).

Although senescent glial cells have been demonstrated to exacerbate pathological changes in neurodegenerative diseases, the role of senescent astrocytes in aging or AD has not been fully characterized yet. Elimination 70%–80% of senescent cells abrogated repressed neurogenesis elicited by PQ‐induced senescence of astrocytes, indicating that senescent astrocytes may impede neurogenesis (Chinta et al., 2018). Senescent astrocytes accumulation promotes insoluble tau aggregates, and drive neurodegenerative disease (Bussian et al., 2018). Interestingly, beta‐amyloid triggered senescence of astrocytes in vitro, which implied mutual promotion of astrocyte senescence and beta‐amyloid deposition (Bhat et al., 2012). Senescent astrocytes express a characteristic of SASP, which in turn could elicit deleterious effects on the surrounding neurons, disturb the maintenance of homeostasis in brain, in large part, contribute to age‐related inflammation and chronic neurodegenerative diseases (Bhat et al., 2012; Han et al., 2020; Hou et al., 2018; Hou et al., 2017; Hou et al., 2019; Salminen et al., 2011; C. Yu et al., 2017). In our study, we found that astrocytic YAP was downregulated in aged mice and AD model mice in a Hippo kinases dependent manner, and deletion of YAP or activation of YAP in astrocytes promotes or delays the aging of the brain, respectively, indicating that senescent astrocytes by YAP downregulation may contribute to aging of the brain and AD.

Several previous studies have revealed that YAP‐CDK6 pathway inhibits cellular senescence (Q. Xie et al., 2013; X. Xu et al., 2020; Yang et al., 2021). Consistent with previous studies, in our studies, several lines of evidences suggest that YAP‐CDK6 signaling mediates D‐gal‐induced senescence of astrocytes. Firstly, YAP was downregulated not only in the hippocampal astrocytes of aging mice and AD model mice, but also in D‐gal and PQ‐induced senescent astrocytes. Secondly, YAP deletion in astrocytes significantly reduced CDK6 expression, aggravated cell senescence, and impaired cognitive function. Thirdly, overexpression of CDK6 partially rescued the senescence of astrocytes, and inhibition of CDK6 abolished XMU‐MP‐1's preventive effects on senescence of astrocytes. Moreover, as shown in Figure S7a–c, the transcription factor FOXM1, which is the substrate of CDK6 and protects cancer cells from senescence (Anders et al., 2011; Rader et al., 2013), showed decreased expression in senescent astrocytes, and another substrate of CDK6, the oxygen sensor PHD1 (Kennel et al., 2018; Ortmann et al., 2016), showed increased expression, indicating that FOXM1 and PHD1 might be the downstream targets of CDK6 during astrocytic senescence. In future, how YAP‐CDK6 signaling prevents astrocytic senescence requires further study.

Some strategies are developed to remove senescent cells relatively specifically and ameliorate the condition of the aging persons, such as the senolytic therapy (combined use of dasatinib and quercetin) (M. Xu et al., 2018; Zhang et al., 2019). In our study, we found that activation of YAP by XMU‐MP‐1 partially improved the cognitive function of AD model mice and old mice, and inhibition of CDK6 promoted astrocytic senescence. These evidences indicated that activation of the YAP‐CDK6 pathway may delay brain aging and aging‐related neurodegenerative diseases. However, a number of studies have demonstrated that YAP and CDK6 promote tumorigenesis (Li et al., 2019; Takeuchi et al., 2017). It is possible that the YAP‐CDK6 pathway may promote tumor progression under excessive activation, and may rejuvenate senescent astrocytes with moderate activation, thereby playing dual roles under cancer or cellular senescence conditions, depending on the activation degree of this pathway, although this remains to be studied further. Therefore, in future, to delay brain aging and aging‐related neurodegenerative diseases, appropriate activation of YAP should be considered carefully, and further investigations are required.

In summary, our studies identified unrecognized functions of YAP in preventing astrocytic senescence in vitro and in vivo, revealed the YAP‐CDK6 pathway in the negative control of astrocytic senescence, and discovered the new function of XMU‐MP‐1 in anti‐aging, which may provide new insights and targets for delaying brain aging and aging‐related neurodegenerative diseases.

## 4 EXPERIMENTAL PROCEDURES

### 4.1 Animals

YAP^GFAP^‐CKO mice were generated by crossing the floxed Yap allele (YAP^f/f^) with GFAP‐Cre transgenic mice (Shanghai Model Organisms), maintained in a C57BL/6J strain background. YAP^f/f^ mice were generated as previously described (Z. Huang et al., 2016; C. Xie et al., 2020). AD model mice (APP/PS1 mice) were purchased from Shanghai Model Organisms, and all mice were confirmed by genotyping. All animals were randomly assigned to experimental groups. Blind evaluation of genotype and experimental condition was performed. The mice were utilized to assess histological, biochemical, and behavioral functions. The use of experimental animals has been approved by Animal Ethics Committee of Wenzhou Medical University.

### 4.2 cDNA constructs

pcDNA3.1‐CDK6 (http://www.addgene.org/75170/) was purchased from Addgene and its sequence was verified by sequencing.

### 4.3 Reagents

D (+) ‐ galactose (D‐gal, D8310, Solarbio) was applied at the concentration of 111 mM. PQ (36541, Sigma) was dissolved in water and was applied at the concentration of 40 μM for 24 h. CP‐10 (HY‐125835, MedChemExpress) was dissolved in DMSO and was applied at 2 nM for 4 h. XMU‐MP‐1 (Y‐100526, MedChemExpress) was dissolved in DMSO and was applied at 5 μM for 24 h, and injected intraperitoneally at a dose of 1 mg/kg (Fan et al., 2016), given every 2 days, and lasted for 2 weeks. All the concentrations of these reagents are indicated above, if there is no special instruction.

### 4.4 Cell culture and transfection

Primary astrocyte cultures were prepared from the cerebral cortex of P1‐P3 mice as described previously (Z. Huang et al., 2016). The purity of GFAP positive (a marker of astrocytes) cells in our culture system was more than 94%. Appropriate plasmids (2 μg per 35‐mm dish) were transfected into the cells using the Lipofectamine™ 3000 Transfection Reagent (L3000‐015, Invitrogen) as the manufacturer's instructions.

### 4.5 Western blotting

Western blotting was carried out as described previously (X. Xu et al., 2020). Primary antibodies used in this study including rabbit anti‐YAP (ab205270, Abcam, 1: 1, 000), mouse anti‐YAP (WH0010413M1, Sigma, 1:1, 000), rabbit anti‐p‐YAP (#13008, CST, 1:1, 000), rabbit anti‐LATS1 (#3477, CST, 1:1, 000), rabbit anti‐p‐LATS1 (ser909) (#9157, CST, 1:1, 000), rabbit anti‐MST1 (#3682, CST, 1:1, 000), rabbit anti‐p‐MST1/2 (Thr183/Thr180) (#49332, CST, 1:1, 000), rabbit anti‐SAV1 (#13301, CST, 1:1, 000), rabbit anti‐p‐MOB1 (Thr35) (#8699, CST, 1:1, 000), rabbit anti‐p53 (bs‐2090R, Bioss, 1:1, 000), rabbit anti‐Lamin B1 (ab16048, Abcam, 1: 1, 000), mouse anti‐CDK6 (#3136T, CST, 1:1, 000), rabbit anti‐FOXM1 (AV39518, Sigma, 1: 1, 000), and mouse anti‐PHD1 (F5303, Sigma, 1: 1, 000). Mouse anti‐β‐actin (A5316, Sigma, 1:10, 000) or rabbit anti‐GAPDH (#2118, CST, 1:5, 000) was used as a loading control. The protein signals were detected using ECL detection kit (Bio‐Rad) and analyzed using Quantity One software (Bio‐Rad).

### 4.6 Immunostaining

For cultured cells, immunostaining was performed as previously described (X. Xu et al., 2020). Primary antibodies included rabbit anti‐YAP (ab205270, Abcam, 1:200), mouse anti‐YAP (WH0010413M1, Sigma, 1:200), mouse anti‐GFAP (MAB360, Millipore, 1:500), and rabbit anti‐Lamin B1 (ab16048, Abcam, 1:200).

For staining of tissue sections, immunostaining was conducted as previously described (C. Xie et al., 2020). Primary antibodies included rabbit anti‐YAP (ab205270, Abcam, 1:200), mouse anti‐YAP (WH0010413M1, Sigma, 1:200), mouse anti‐GFAP (MAB360, Millipore, 1:500), mouse anti‐NeuN (ab177487, Abcam, 1:500), goat anti‐Iba1 (ab5076, Abcam, 1:500), rabbit anti‐Lamin B1 (ab16048, Abcam, 1:200), and rabbit anti‐Ki67 (AB9260, Millipore, 1:200). Mounting was done after another three washes. Images were acquired by using a fluorescence microscopy or a confocal microscopy (Zeiss) and analyzed by Image J software.

### 4.7 Quantitative real‐time PCR (qRT‐PCR)

Total RNA extraction, reverse transcription and the quantification of mRNA levels were done as previously described (X. Xu et al., 2020). β‐actin or GAPDH was chosen as the endogenous control. The fold change of gene expression was calculated using the 2^−ΔΔCt^ method. The primers were synthesized by Sangon Biotech and presented as follows: *p16*, 5’‐AATCTCCGCGAGGAAAGC‐3’, 5’‐GTCTGCAGCGGACTCCAT‐3’ (Chinta et al., 2018); *p21*, 5’‐TGTCGCTGTCTTGCACTCTG‐3’; 5’‐GACCAATCTGCGCTTGGAGT‐3’ (Cheng et al., 2017); IL‐6, CTGCAAGAGACTTCCATCCAG, AGTGGTATAGACAGGTCTGTTGG (Wang et al., 2018); IL‐8, CCTAGGCATCTTCGTCCGTC, CAGAAGCTTCATTGCCGGTG (Wei et al., 2019); *MMP*‐*3*, 5’‐ AGGGATGATGATGCTGGTATG‐3’, 5’‐AACACCACACCTGGGCTTAT −3’ (M. Chen et al., 2016); *CDK6*, 5’‐GAGTGTCGGTTGCATCTTT‐3’, 5’‐GAGTCCAATGATGTCCAAGA‐3’ (Mi et al., 2018); LATS1, AAAGCCAGAAGGGTACAGACA, CCTCAGGGATTCTCGGATCTC (Wang et al., 2018); MST1, CTCACCACTGAATGACTTCCAG, AAGGCCCGACAGTCCAGAA (Millar et al., 2020); SAV1, GTCATCCCCTTGAACGAGAA, ATACCACTGCTGCCTCTGCT (Q. Chen et al., 2014); *GAPDH*, TGCACCACCAACTGCTTAG, GGATGCAGGGATGATGTTC (Benrick et al., 2017); *β*‐*actin*, 5’‐GGCTGATTCCCCTCCATCG‐3’, 5’‐CCAGTTGGTAACAATGCCATGT‐3’ (Y. Shen et al., 2014).

### 4.8 Induction of astrocyte senescence

Astrocyte senescence models were established by either D‐gal (Y. Shen et al., 2014), or PQ (Chinta et al., 2018) as described previously, with slight modifications. Briefly, astrocytes at DIV 7–11 were replanted onto poly‐D‐lysine‐coated dishes with the same culture medium supplemented with 111 mM D‐gal for another 5 days (Y. Shen et al., 2014). For PQ‐induced senescence of astrocytes, astrocytes were treated with 40 μM PQ for 24 h and recovered for another 8 days (Chinta et al., 2018).

### 4.9 SA‐β‐gal staining

SA‐β‐gal staining kit (G1580, Solarbio) was adopted to assess cellular senescence according to the protocol clearly provided by the manufacturer, as previously described (X. Xu et al., 2020).

### 4.10 Spatial reference memory on the elevated Y‐maze

The Y‐maze task for hippocampus‐dependent spatial reference memory was performed as previously described (Zhang et al., 2019), with slight modifications. Briefly, mice were habituated in the testing room overnight before the spontaneous alternation test by using an opaque perspex Y‐maze (40 cm long and 9 cm wide with 21 cm high walls). Each animal was placed in turn in arm A of the Y‐maze and allowed to explore for 8 min, and the arm entries and walking distance were recorded. All equipment was cleaned between each trial with 75% ethanol. Spontaneous alternation was defined as a successive entry into three different arms, on overlapping triplet sets. The maximum number of alternations was defined as the total number of arm entries minus 2. The percentage number of alternations (alternation %) = the number of actual alternations/the maximum number of alternations ×100%, which was an indication of cognitive function. All behavioral observations were made between 15:00 and 18:00 pm. The number of actual alternations and the maximum number of alternations were recorded.

### 4.11 Barnes circular maze

The Barnes circular maze test was performed on a white circular surface, 92 cm in diameter, with 20 holes equally spaced around the perimeter as previously described (Nanou et al., 2016), with slight modifications. A black Plexiglas escape box (15 × 7 × 7 cm) containing paper cage bedding on its floor was located under one of the holes (defined as the target hole) in a room with a light intensity of 1200 lux. The location of the target was consistent for a given mouse. Firstly, adaptation was accomplished by each mouse. For each mouse, it was guided into the hole twice within 3 min, at an experiment interval of 15 min. Training lasted for 4 successive days (day 1–4) were performed, each mouse for 2 trials, with a 15 min interval. To start a trial, a test mouse was placed in the center of the maze, held there for 10 s by using a dark box, and then the mouse was allowed to explore freely. Once the mouse entered the escape box, it was remained undisturbed for 1 min with the light off. Mice that failed to find the target hole were gently guided to the escape box. On the fifth day (day 5), a probe trial was conducted. The escape box was changed to another hole and the maze was rotated to keep the spatial position of the target hole unchanged, in order to avoid odor guidance. All equipment was cleaned between each trial with 75% ethanol. Time latency to reach the target hole and the search errors were recorded.

### 4.12 Statistical analysis

All data values were expressed as mean ± SEM derived from at least three independent experiments. GraphPad Prism software was used for statistical analysis. Student's *t*‐test and ANOVA analysis were applied in this study. A *p* value of <0.05 was considered to be statistically significant.

## CONFLICT OF INTEREST

The authors declare no competing interests.

## AUTHOR CONTRIBUTIONS

Z.Z.H and X.X.X contributed to the conception and design of the study. X.Y.S and X.X.X did most of western blot and immunofluorescent staining experiments. J.J.W and M.X.W was mainly responsible for the cell culture experiments, and X.Y.S and X.X.X both contributed to the acquisition and interpretation of the data. W.J.F, J.J.W, X.M.M, Q.W, X.N.W, L.H.W, Y.M.M, S.W and X.M.B, helped to extract the plasmids and analyze some of the staining data. X.M.B and J.J.W also contributed to mouse breeding and genotyping. X.X.X and W.J.F wrote the manuscript. X.X.X, W.J.F, Y.W, and Z.Z.H contributed to revising the original text.

## Supporting information

Fig S1

Fig S2

Fig S3

Fig S4

Fig S5

Fig S6

Fig S7

## Data Availability

The authors declare that all data supporting the conclusions of this study are presented within the paper and the supplementary information files and are available from the authors.
