# Supplementary figures and images for: YAP prevents premature senescence of astrocytes and cognitive decline of Alzheimer's disease through regulating CDK6 signaling
Source: Aging Cell. 2021 Aug 20;20(9):e13465. doi: 10.1111/acel.13465 (PMC8441453; doi:10.1111/acel.13465)

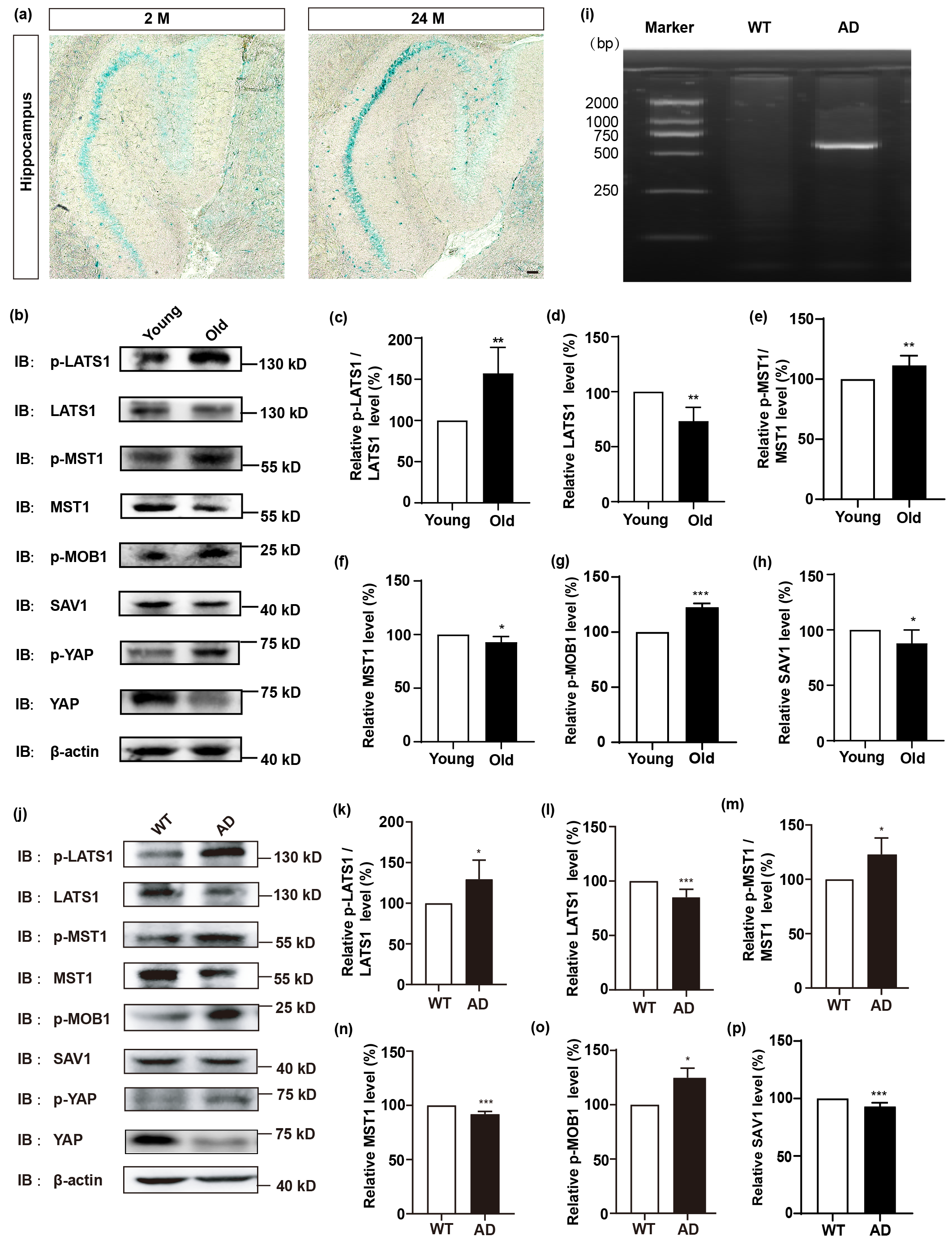

Supplement: Supplementary file 1 — Fig S1 [file ACEL-20-e13465-s007.tif]

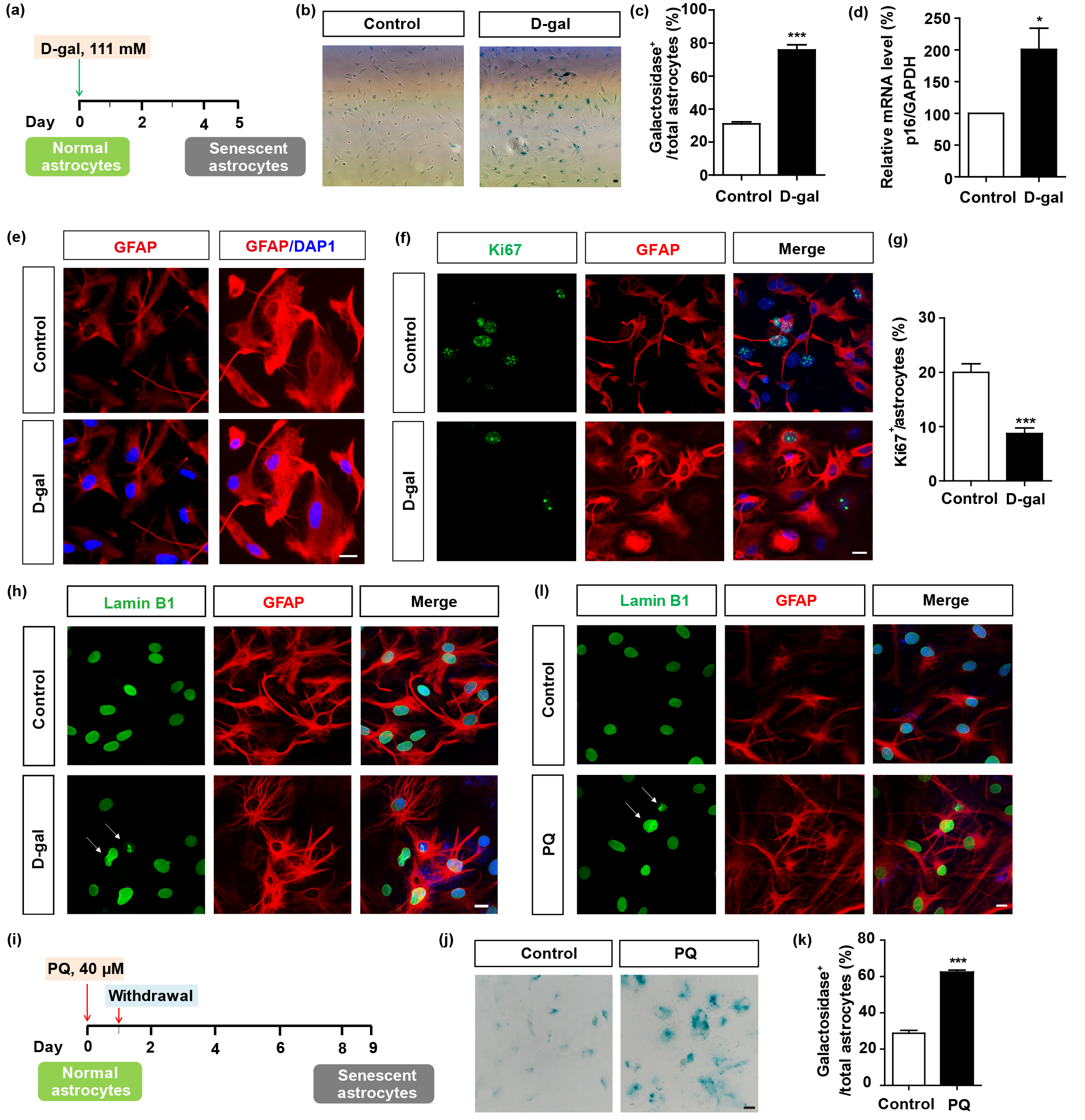

Supplement: Supplementary file 2 — Fig S2 [file ACEL-20-e13465-s003.tif]

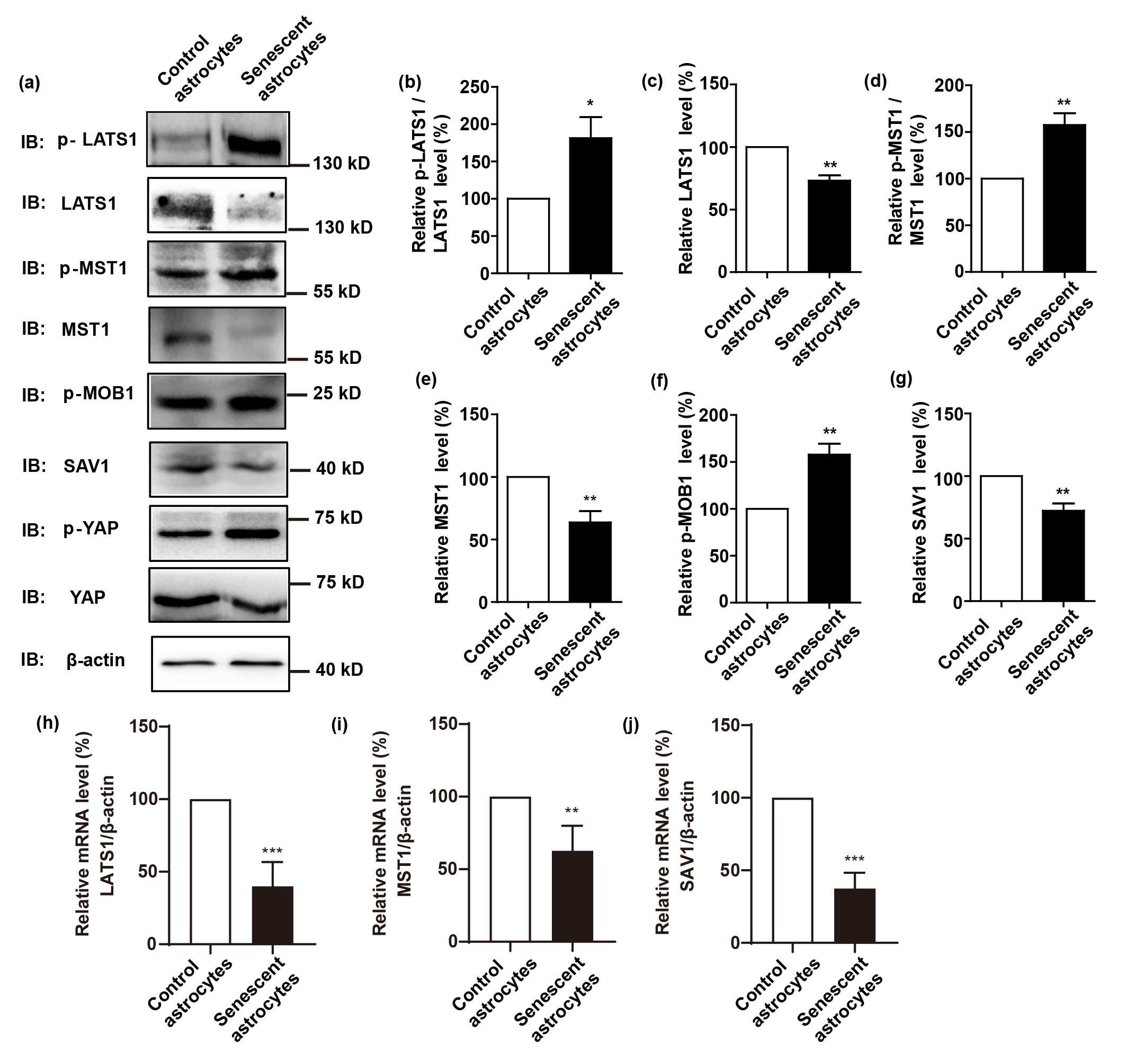

Supplement: Supplementary file 3 — Fig S3 [file ACEL-20-e13465-s005.tif]

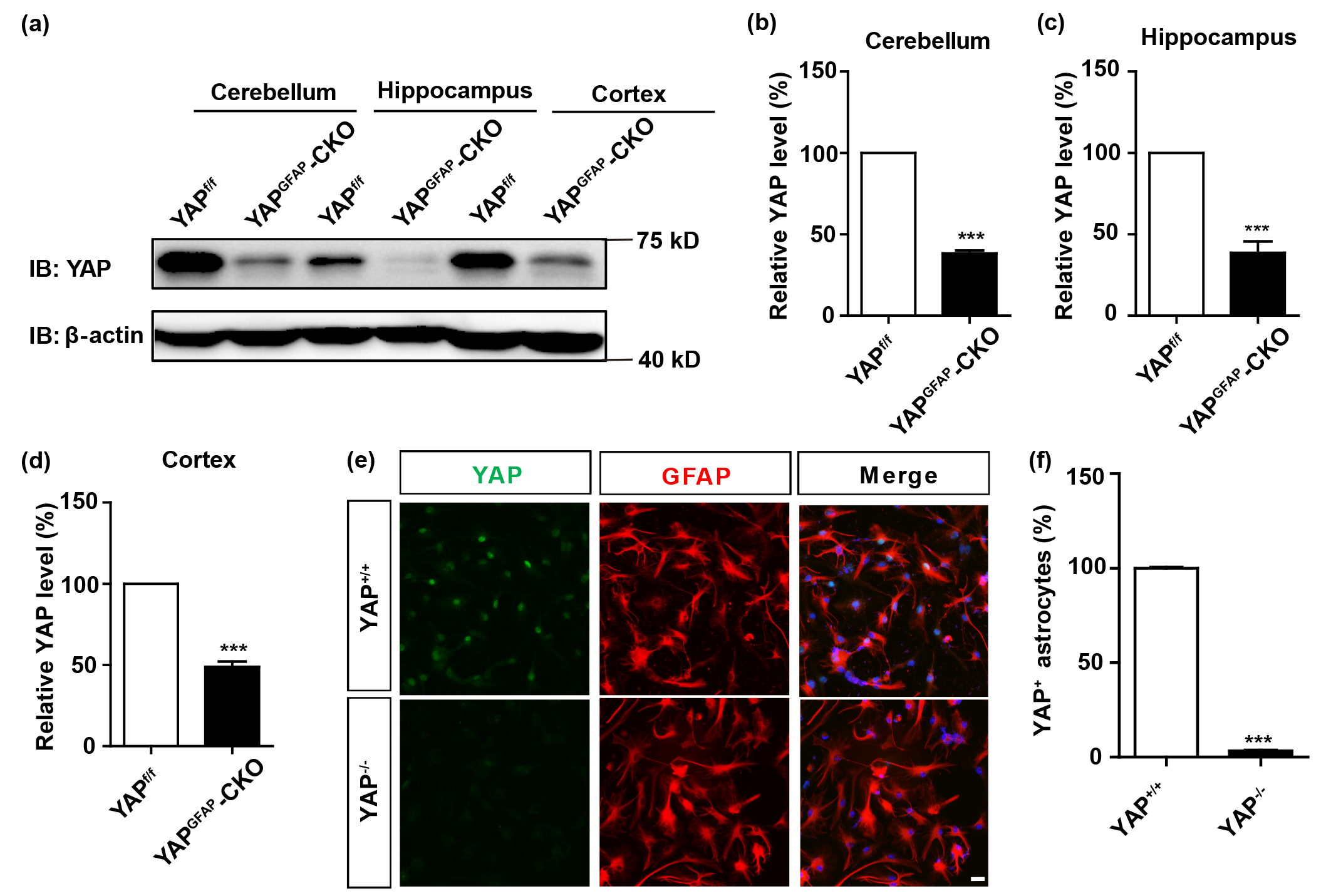

Supplement: Supplementary file 4 — Fig S4 [file ACEL-20-e13465-s004.tif]

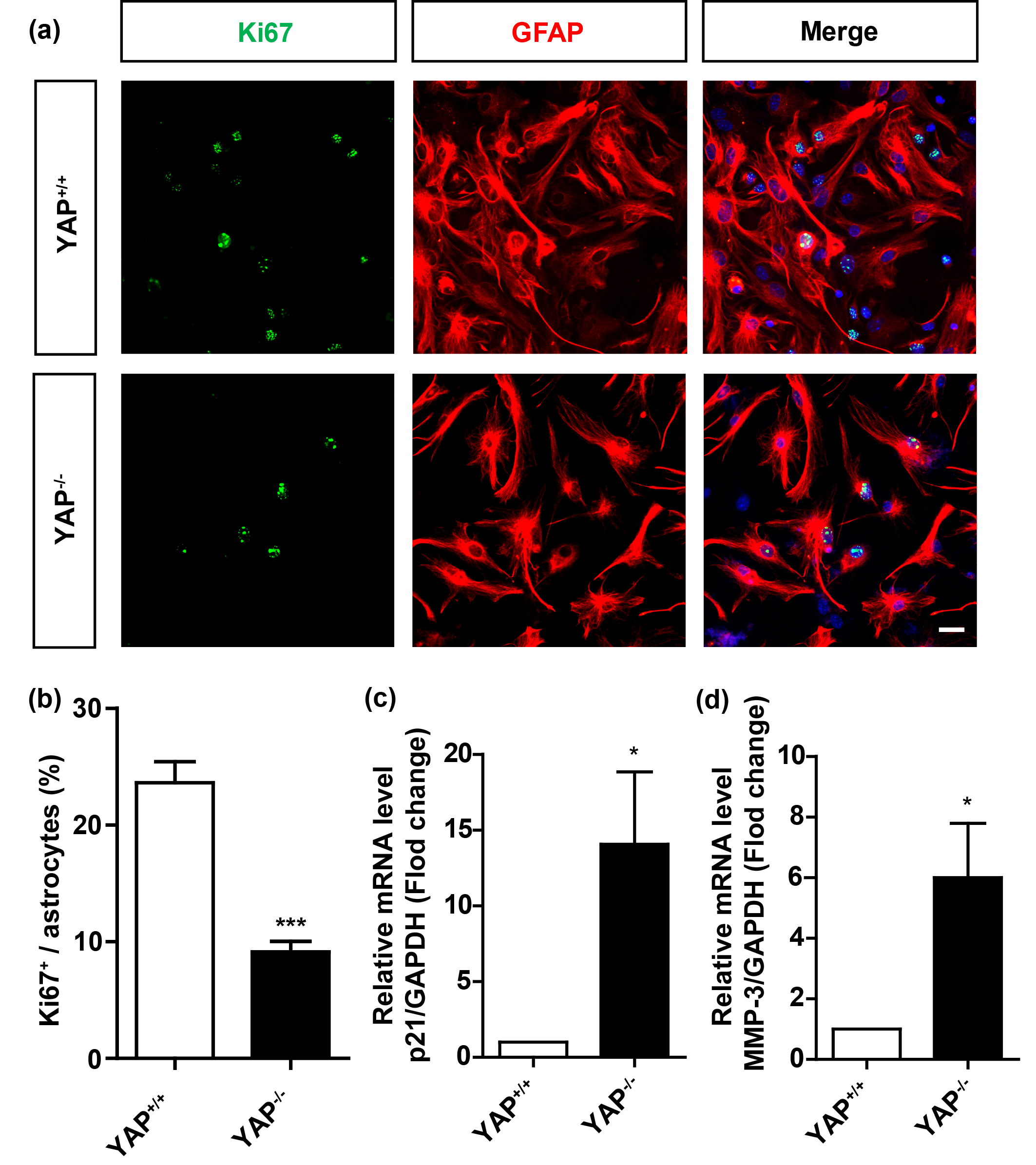

Supplement: Supplementary file 5 — Fig S5 [file ACEL-20-e13465-s006.tif]

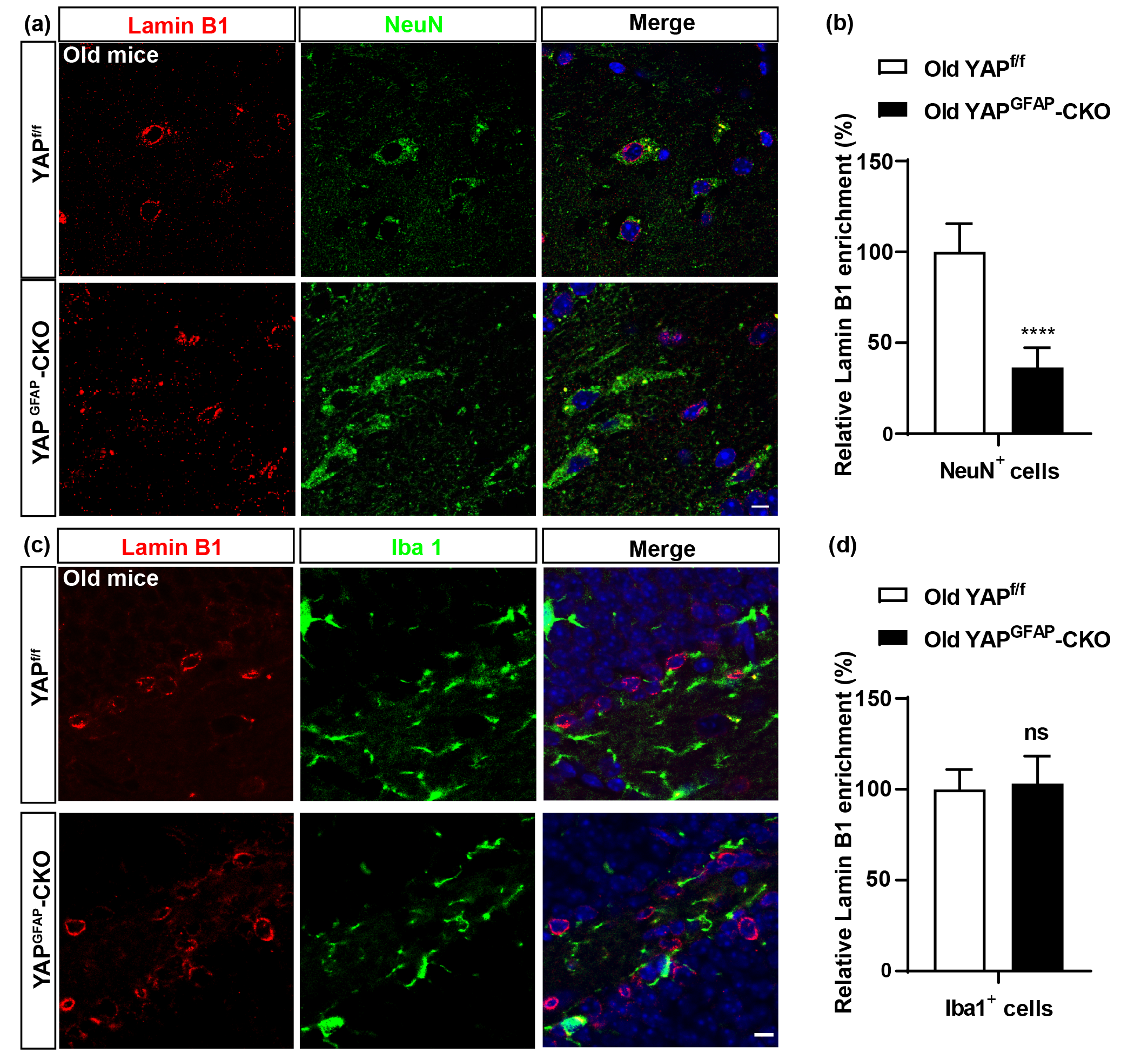

Supplement: Supplementary file 6 — Fig S6 [file ACEL-20-e13465-s002.tif]

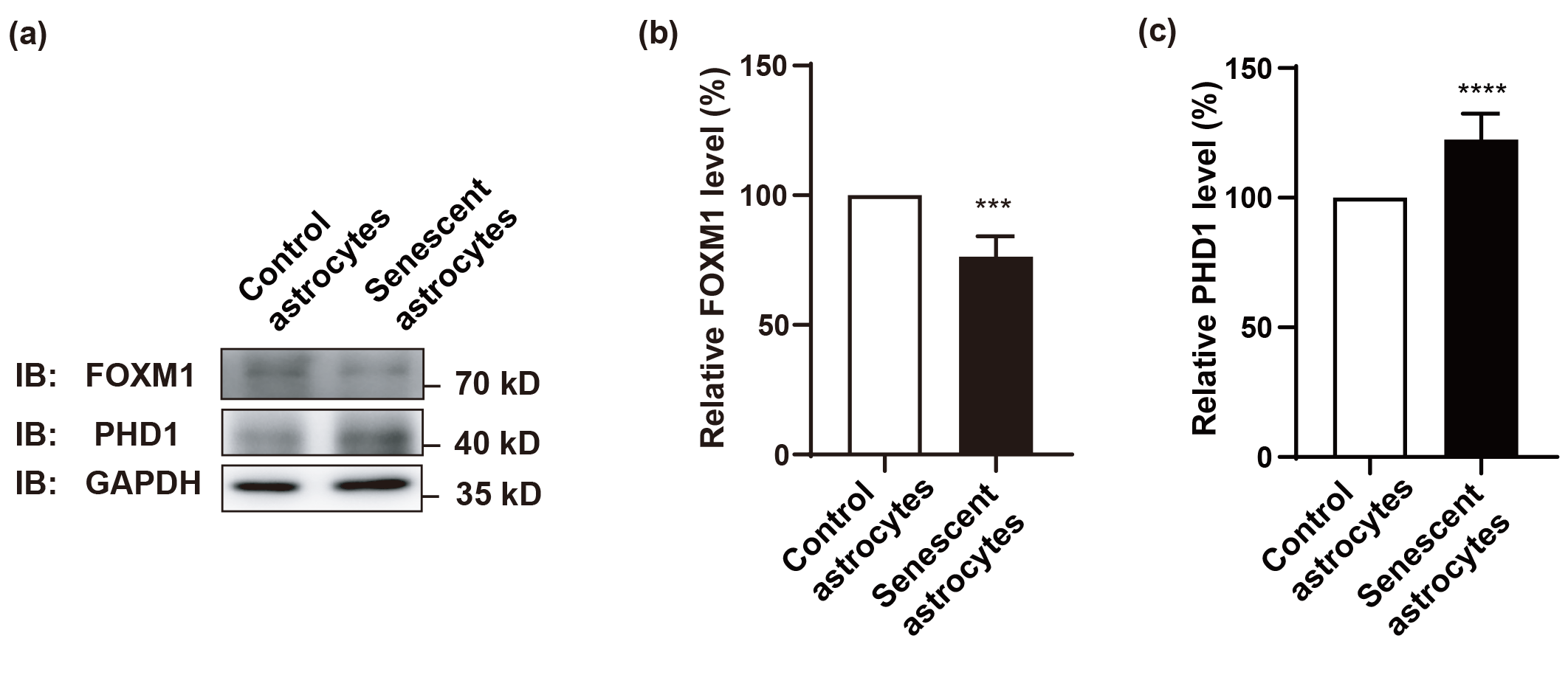

Supplement: Supplementary file 7 — Fig S7 [file ACEL-20-e13465-s001.tif]
